# The working memory costs of a central attentional bottleneck in multitasking

**DOI:** 10.1007/s00426-021-01615-1

**Published:** 2021-11-09

**Authors:** Pauldy C. J. Otermans, Andrew Parton, Andre J. Szameitat

**Affiliations:** Division of Psychology, Department of Life Sciences, Brunel University London, Kingston Lane, Uxbridge, UB8 3PH UK

## Abstract

When two (or more) tasks, each requiring a rapid response, are performed at the same time then serial processing may occur at certain processing stages, such as the response selection. There is accumulating evidence that such serial processing involves additional control processes, such as inhibition, switching, and scheduling (termed the active scheduling account). The present study tested whether the existence of serial processing in multitasking leads to a requirement for processes that coordinate processing in this way (active scheduling account) and, furthermore, whether such control processes are linked to the executive functions (EF) of working memory (WM). To test this question, we merged the psychological refractory period (PRP) paradigm with a WM task, creating a complex WM span task. Participants were presented with a sequence of letters to remember, followed by a processing block in which they had to perform either a single task or a dual task, and finally were asked to recall the letters. Results showed that WM performance, i.e. the amount of letters recalled in the correct order, decreased when performing a dual task as compared to performing a single task during the retention interval. Two further experiments supported this finding using manipulations of the dual task difficulty. We conclude that the existence of serial processing in multitasking demands additional control processes (active scheduling) and that these processes are strongly linked to the executive functions of working memory.

## Introduction

An important aspect of everyday life is the ability to perform two tasks at the same time, so-called dual-task or multitasking performance. However, people are frequently unable to perform certain mental operations in parallel, resulting in slowed response times and/or increased error rates when performing dual-tasks as compared to single-tasks (Pashler, 1994). There is strong evidence for a central attentional bottleneck in dual-task performance (Marois & Ivanoff, 2005; Pashler, 1994; Tombu et al., 2011) that causes certain mental operations to be processed serially. There is accumulating evidence (Koch et al., Kübler et al., 2018; Marois & Ivanoff, 2005; Schubert, 2008; Tombu et al., 2011) that this serial processing is linked to executive functions (EF), for instance to schedule the order in which the tasks are processed, to inhibit tasks, and to switch between tasks The aim of the current paper is to characterize the nature of these EFs and in particular elucidate their link to the EFs proposed in the context of working memory (WM), i.e. whether they are a related or independent set of mental functions.

## The central attentional bottleneck

**Fig. 1:**
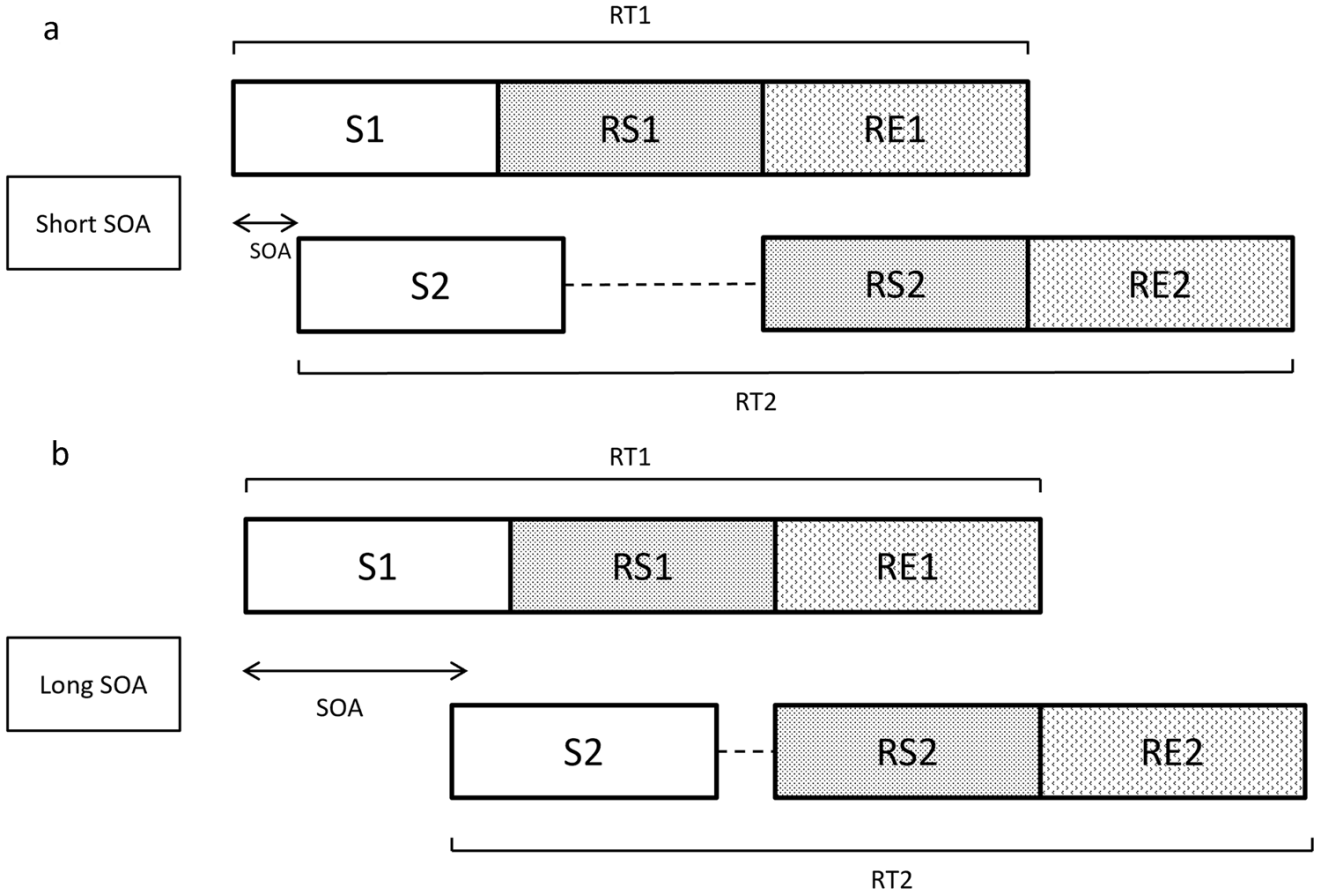
The response-selection theory of the Psychological Refractory Period (PRP) paradigm **a** short stimulus-onset asynchrony (SOA). **b** long SOA. The dashed line indicates the PRP

A prototypical paradigm for investigating the central attentional bottleneck is the Psychological Refractory Period (PRP). In this paradigm, two stimuli (S1 and S2) each requiring a specific response (R1 and R2) are presented either simultaneously or after one another separated by a (variable) stimulus-onset asynchrony (SOA) (De Jong, 1995); Fig. 1.

When the two tasks (Task 1 and Task 2) have to be performed simultaneously [Stimulus Onset Asynchrony (SOA), 0 ms] or rapidly after each other (SOA > 0 ms), research has shown that processing of response selection can only work serially, i.e. it constitutes a processing bottleneck (Pashler, 1994). As a consequence, the response selection of Task 2 has to wait until the response selection of Task 1 has finished (termed the refractory period). In contrast, peripheral stages, such as perception and motor execution, have been shown to mostly work in parallel.

The typical finding in this paradigm is a prolonged response time to S2 (RT2) in dual-task conditions, as compared with presenting S2 in isolation. In addition, RT2 increases with decreasing temporal overlap between the tasks (i.e. shorter SOA between S1 and S2) whereas RT1 is usually largely unaffected by the SOA (De Jong, 1995; Lee & Chabris, 2013; Logan & Gordon, 2001; Marois et al., 2006; Pashler, 1994; Smith, 1967). This prolongation of RT2 with shorter SOAs is a phenomenon often referred to as the PRP effect and is a consequence of the required serial processing. If the SOA is short, the second task has to wait until processing of the first task has finished at the serial processing stage. However, for longer SOAs processing of task 1 has progressed further when task 2 arrives at this serial stage, and, consequently, the waiting time (PRP) is shorter. It is a highly robust effect which has been observed in a wide variety of tasks (including simple RT and choice RT tasks), response modality combinations (e.g. vocal–manual, foot–manual, or manual–manual), and combinations of stimulus modalities (e.g. visual–visual or visual–auditory) (Koch et al., 2018; Pashler, 1990, 1994; Tombu & Jolicœur, 2004). The current study used the PRP paradigm in combination with a WM task and is explained in detail later.

The existence of the PRP effect can be taken as an indication of serial processing. A processing stage working serially has frequently been termed bottleneck and we will adopt this terminology but without any assumptions regarding its theoretical underpinnings (e.g. whether the bottleneck is structural or strategic, see further below). Previous research has aimed to identify where in the cognitive architecture this bottleneck is localized. Cognitive models of choice-response tasks often assume the presence of at least three processing stages; perception, response selection and motor execution (Pashler, 1994). Research has shown that for a particular task each stage has to be completed before the next stage can commence (Pashler, 1993). However, across different tasks, perception and motor execution can work in parallel while the response selection can only be processed serially for one task at a time (Pashler, 1993). As a consequence, response selection for stimulus 2 (RS2) has to wait until response selection for stimulus 1 (RS1) is finished. While most research indicates that a bottleneck occurs at the response selection stage, there is still some debate about whether other processing stages may result in bottlenecks (Dux et al., 2006; Koch et al., 2018; Marois & Ivanoff, 2005; Pashler, 1994; Sigman & Dehaene, 2005; Spence, 2008; Szameitat et al., 2006). However, for the current paper, only the presence of a bottleneck, as indicated by the presence of a PRP effect, is of relevance, but not at which processing stage(s) it is located.

## The central attentional bottleneck and executive functions

When two tasks are processed serially at a processing stage, a crucial problem arises: In which order should the tasks be processed? This is particularly relevant in PRP tasks, because participants are typically instructed to respond to the tasks in a given order. Previous research has overwhelmingly shown that participants have voluntary control over the processing order of the tasks, refuting a simplistic first-come first-served account (DeJong, 1995; Fischer & Plessow, 2015; Luria & Meiran, 2003; Schubert, 2008; Szameitat et al., 2006).

For example, De Jong (1995) investigated the role of preparation on overlapping task performance. In his study, two two-choice-response tasks were performed in a PRP dual-task condition. As typical in the PRP paradigm, the participants were instructed to process the tasks in the order of their presentation. However, De Jong varied the task-order from trial-to-trial, which required participants to also adjust their response order across individual trials. This manipulation showed that participants explicitly plan and prepare in advance in which order to process the tasks. Specifically, participants automatically prepare to process the tasks in the same order as they did in the previous trial. However, participants are nonetheless able to override this initial preparation if the task order changes unpredictably. De Jong (1995) concluded that additional control processes are needed to coordinate task processing in the PRP dual-task which are not required for either task alone. Luria and Meiran (2003) confirmed De Jong's (1995) results and furthermore showed that at least some of these control processes work on central stages in the task processing and not only on the coordination of the motor hand sequences.

**Fig. 2:**
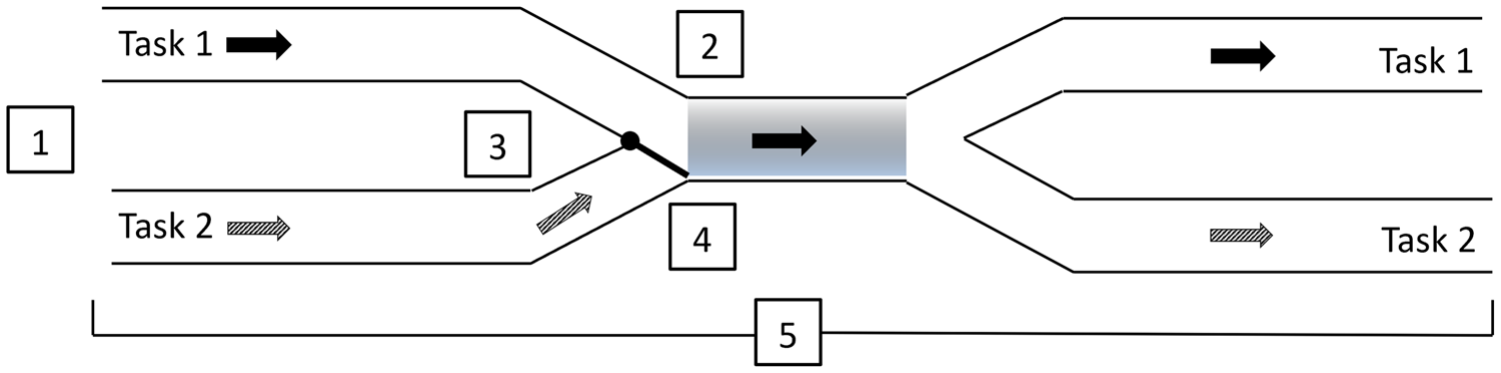
Simplified representation of the bottleneck in multitasking. Numbers denote potential executive functions. 1. Sequencing the processing order of the tasks. 2. Switching the bottleneck mechanism between tasks. 3. Inhibition of the second task while the bottleneck is occupied by the first task. 4. Activation to reengage second-task processing once bottleneck is free. 5. Monitoring for correct processing and potential interference

The role of such control processes depends on the underlying theoretical model of task processing in PRP dual-tasks. While it is largely undisputed that serial processing virtually always occurs in PRP dual-tasks, there is less consensus on its cause. Proponents of a structural bottleneck claim that the serial processing stage(s) simply cannot work in parallel; therefore, serial processing is inevitable and the bottleneck itself is considered to be immutable. Under this proposal, it has been suggested that EFs coordinate the processing of the tasks at the bottleneck to avoid interference. Thus, the view is that there is an immutable structural bottleneck and, consequently, EFs are required. In more detail, it has been suggested that when the bottleneck is actively switched to the first task, the second task is inhibited so that it does not enter the bottleneck and causes interference while the bottleneck is processing the first task. Then, the bottleneck is switched to the second task when processing the first task is finished, and finally second task processing will be re-instantiated (Meyer & Kieras, 1997; Schubert, 2008; Szameitat et al., 2006); Fig. 2.

The main alternative model is that the serial processing does not occur because of a structural bottleneck, but because of a processing strategy. For example, capacity sharing models assume that mental resources can be gradually shared between the tasks, so that in principle both tasks could be processed fully in parallel, each receiving 50% of the available resources. However, theoretical modelling (Executive Control of Visual Attention, ECTVA model) has shown that this is actually sub-optimal, because there is potential cross-talk between the tasks during response selection. This relates to a difficulty in attributing the correct stimuli and responses to each task, which has been called the ‘dual-task binding’ problem (Lehle & Hübner, 2009; Logan & Gordon, 2001). This problem is automatically resolved when the tasks are processed serially, i.e. by first assigning 100% of the resources to the first task and subsequently 100% of the resources to the second task. The proposed processes are surprisingly similar to those proposed by the structural bottleneck model, i.e. scheduling the correct processing order by inhibition of the second task, switching between the tasks, and reinstating the second task by means of multiple consecutive re-assigning of the resources. Here, the view would be that a bottleneck appears to be present because of EFs which schedule the tasks serially. In summary, a strategic bottleneck implies that the bottleneck is a consequence of EF, while a structural bottleneck implies that the EF are demanded to resolve the consequences of the bottleneck.

It is worth noting that the functions required by both models are quite similar and are all prototypical key functions of the so-called EF of WM (Baddeley & Hitch, 1974; Engle et al., 1999; Miyake et al., 2000). However, the human-action focused research into the PRP paradigm and the memory focused research into EF in WM have, to our knowledge, not been integrated. Therefore, it is conceivable that, despite similar terminology, inhibition of a task set in the PRP paradigm is different to inhibition of memory contents in WM tasks, i.e. they are distinct mental functions which have been given the same name by two distinct research communities. Alternatively, it might be that both instances actually refer to the same mental process, i.e. there is only one inhibition function for both, the inhibition of task sets and the inhibition of short-term memory contents. Therefore, our aim was to determine whether the processes involved in coordinating serial processing are related to the EF of WM.

## Executive functions of working memory

Working memory is often defined as the short-term storage of information (maintenance) plus executive functions (Baddeley & Hitch, 1974). In some models, the ‘executive function’ component is differently named, (e.g. controlled attention) but its function remains virtually identical (Engle, 2002). This executive system enables the manipulation of the contents of short-term memory (Baddeley & Hitch, 1974). The most prototypical functions to exert this control on memory contents and other cognitive processes are inhibition, switching, and updating (Miyake et al., 2000).

In the WM literature (which is surprisingly separate from the literature on central attentional bottlenecks and the PRP paradigm), numerous WM models have been proposed. However, many of them are very specific to memory processes and are not easily linked to the demands on controlled attention arising from processing a task investigating human action performance, such as the PRP task. One of the few exceptions is the time-based resource sharing (TBRS) model proposed by Barrouillet and Camos (2007), which makes very strong and testable predictions about the interactions between memory-related and task-related processing demands. Accordingly, the current study employed the TBRS model as conceptual framework for WM.

## Time-based resource sharing (TBRS) model

Like most other WM models, the TBRS model proposes that WM consists of short-term storage (maintenance) and controlled attention (manipulation, EF) (Barrouillet & Camos, 2007). However, it also specifies the time course and demands of the different processes in a high level of detail. This applies in particular to so-called complex WM span tasks, in which a short-term memory task (e.g. remembering a set of letters) is combined with an independent processing task (e.g. solving math equations, such as in the operation span task; Unsworth et al., 2005). In the current study, we created a complex WM task by combining a short-term memory task with a PRP dual-task.

The TBRS model is based on four proposals (Barrouillet & Camos, 2007). First, it is assumed that both maintenance and processing of information require the same attentional resource, which is termed controlled attention (Barrouillet & Camos, 2010; Engle et al., 1999; Oberauer & Lewandowsky, 2013). When processing information, controlled attention is required for inhibition of irrelevant and selection of the relevant information, activation of goals, retrieval of information from memory, selection of appropriate responses and monitoring of information. Thus, in the TBRS, controlled attention effectively corresponds to EF (Baddeley, 1996; Engle, 2002; Miyake et al., 2000).

Second, controlled attention is not shared between tasks, but instead it is always fully devoted to one task in an all-or-nothing fashion. According to the TBRS, if a task demands more attentional resources, i.e. has a higher cognitive load, this task occupies controlled attention for a longer period of time. Consequently, less time is available to refresh memory traces in short-term memory, and short-term-memory capacity decreases.

Third, activated memory traces suffer from a time-related decay when attention is switched away. Attention is needed to refresh the memory traces and keep information in memory active and up to date. As a consequence, when attention is switched to another task (since it can only focus on one task at a time) recall in WM tasks will decrease (Barrouillet & Camos, 2010). Variations of this model have been proposed which suggest that memory traces do not necessarily decay per se, but instead deteriorate due to interference (Oberauer & Lewandowsky, 2013). However, this distinction has no implications for the current study.

Fourth, related to the third point, if controlled attention is disrupted or distracted while engaged in the updating required for memory maintenance, memory recall declines. Such disruptions occur in complex WM span tasks, when an additional processing task requires controlled attention, which is also required by another task for memory maintenance. This assumption is crucial to the current study.

The TBRS model predicts information loss by decay. Souza and Oberauer (2015) agree with the TBRS model but predict information loss by reduced temporal distinctiveness, i.e. the relative spacing of events in time determines the degree of interference. To summarize, the TBRS model assumes that the two functions of WM, processing and maintenance, both demand controlled attention. The model further posits that this controlled attention is a unitary resource similar to traditional conceptualizations of EF. Therefore, TBRS predicts that memory maintenance is subject to interference from concurrent task processing (Barrouillet & Camos, 2010).

## The present study

The current study investigates whether the processes needed to coordinate the serial processing in the PRP paradigm are related to the EF of WM. To answer this question, we used the predictions made by the TBRS model and created a complex WM span task, in which the demands of a short-term memory task (maintenance) and a PRP dual-task (processing) can be combined [cf. Liefooghe et al. (2008) for a highly similar approach in the context of the task switching paradigm].

In more detail, we manipulated the nature of the processing tasks performed alongside the memory task by having simpler tasks (e.g. single-task performance) and more complex tasks (e.g. dual-task performance). In this example (Experiment 1), if dual-task performance places greater demands on EF of WM resources than the single tasks, then the TBRS predicts lower memory recall in the dual-task as compared with the single-task condition. This is because the EF related to processing the dual-task (which are not present in the single-task) occupy controlled attention for a longer period of time, leaving less time for the rehearsal of the memory items. Experiments 2 and 3 follow the same logic but investigate the executive demands of the PRP task in more detail using more fine-grained parametric manipulations of the processing task.

The employed experimental logic uses recall performance in the short-term memory task to infer the controlled attention demands of the different processing tasks. For this logic to be valid, it is important that participants do not trade off performance in one task for performance in the other task. For instance, a participant may sustain very high performance in the memory task by performing very poorly in the processing task. To avoid this, participants received frequent feedback on their performance in the processing task and were told to be more accurate in case their accuracy dropped below 80%. As a consequence, differences in demands on controlled attention should show in recall performance.

## Experiment 1

The aim of Experiment 1 was to test whether PRP dual-tasks demand the executive functions (EF) of working memory (WM). For this, participants had to perform a complex WM span task in which they were presented a series of letters to remember, and then performed a processing task during the retention interval, before finally recalling the letters in the serial order of their presentation. The processing task was either a cue-guided PRP dual-task or the individual single-tasks of which the dual-task was comprised. We hypothesized that memory recall is lower in the dual task than the single tasks, which would support the assumption that PRP dual-tasks place higher demand on the EF of WM.

### Method

#### Participants

Formal power calculations using G*Power software were based on the number of participants needed to detect a difference between the recall performance in the single task versus the dual-task conditions. With an alpha level of 0.05, and assuming a medium effect size (*d* = 0.50), these calculations showed that with a minimum of 27 participants, we would have 80% power to detect a true effect. We chose a medium effect size (*d* = 0.50) because this is in the range of effect sizes reported by Liefooghe et al. (2008). Due to pragmatics in participant bookings (overbooking due to expected cancellations), we eventually tested 30 participants in Experiment 1, 30 in Experiment 2 and 34 in Experiment 3. Thirty participants (mean age: 22 years, SD = 3.1, range 18–31 years, 19 female) took part in this Experiment after giving written informed consent. The study was approved by Brunel University’s College of Health and Life Sciences Ethics Committee and participants received £8 for participation.

#### Tasks

**Fig. 3:**
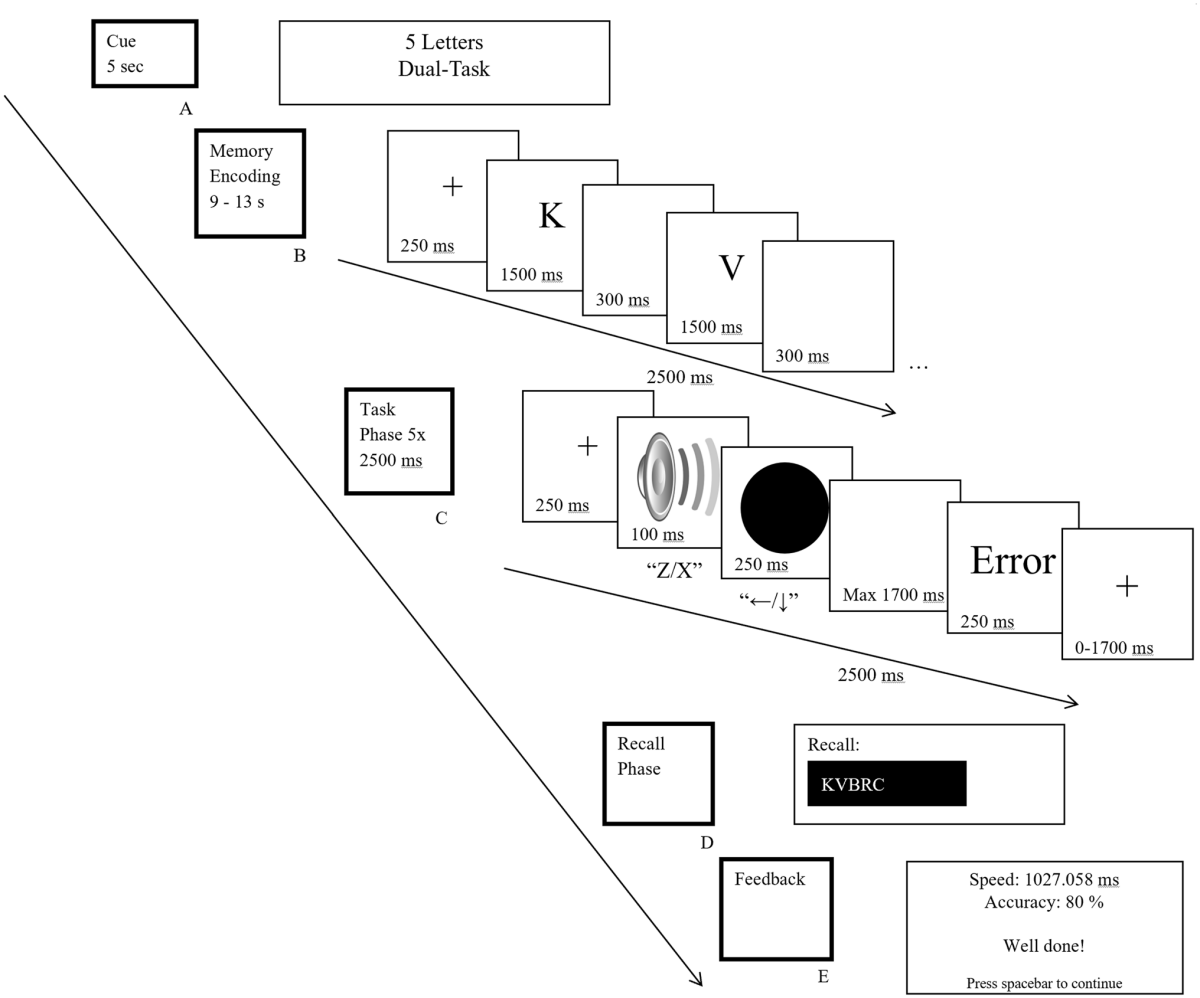
Experimental design of the experiment. A memory trial consisted of five successive phases: A. Cue. B. Memory encoding. C. Task phase (five trials of fixation cross—target(s)—response—feedback each). D. Recall phase. E. Feedback

We used a complex WM span task with preload procedure in which each memory trial consisted of the following phases: block cue, memory-encoding phase, retention interval (auditory single-task, visual single-task, or dual-task), recall phase, and feedback; Fig. 3.

##### Block Cue

At the start of each memory trial, a written instruction was displayed in the centre of the screen for 5 s informing the participants about the memory load and the upcoming processing task, e.g. “6 letters – Dual-task”. This block cue was used to avoid the participants investing mental effort into speculating (at an early stage of the trial) and identifying (at a later stage) which memory and task condition would be presented in the current trial, which might have negatively affected task performance.

##### Memory-encoding phase

In the memory-encoding phase, participants were asked to memorize a series of letters presented sequentially on the screen. These letters had to be recalled in the correct order at a later stage of the memory trial. The letters ‘W’ and ‘H’ were excluded from the set because their pronunciation in English takes longer than other letters of the alphabet (Crannell & Parrish, 1957). Also, the letters ‘T’, ‘P’ and ‘M’ were excluded due to their phonologically similarity to the letters ‘D’, ‘B’ and ‘N’, respectively (Bavelier et al., 2006). Lastly, the vowels (‘A’, ‘E’, ‘I’, ‘O’ and ‘U’) and the letter ‘Y’ were excluded to make sure that the letters to be recalled could not form (pseudo-)words. Therefore, the letters that were included in this experiment were: ‘B’, ‘C’, ‘D’, ‘F’, ‘G’, ‘J’, ‘K’, ‘L’, ‘N’, ‘Q’, ‘R’, ‘S’, ‘V’, ‘X’ and ‘Z’. The letters in the encoding phase were drawn randomly from the set without replacement. Three different memory loads (5, 6, or 7 letters) were used. The memory-encoding phase started with a fixation cross-presented in the centre of the screen for 500 ms. Then a letter was presented for 1500 ms, followed by a 300 ms blank. All subsequent letters were presented individually for 1500 ms with a blank interval of 300 ms between them until the end of the memory load (5, 6 or 7 letters) was reached. Participants were informed that covert rehearsal is allowed, i.e. rehearsing in their head but they were instructed not to whisper or repeat out loud the letters. The experimenter paid close attention to the behaviour of the participant in the practice and no participant attempted visible overt rehearsal.

##### Retention interval

A 12.5 s lasting retention interval followed the memory-encoding phase. During this time, whilst covertly retaining the letters in memory, participants performed one of the three different processing tasks, i.e. auditory single-task, visual single-task, or dual-task.

##### Recall phase

After the retention interval (during which the processing task was performed), participants had to recall the memorized letters in the order they were presented by typing them on a standard UK-English 104-keys computer keyboard. Typed letters were presented on the screen and participant could correct themselves. If a letter was not remembered, participants were instructed to press the spacebar and leave a blank space for the letter. No feedback on recall performance was given.

##### Feedback

To ensure that participants engaged in the processing task during the retention interval, they received feedback on their performance in the processing task at the end of each memory trial. This feedback aimed at keeping accuracy above 80% and the response times below an individual threshold determined during the practice period. If people failed to meet the performance criteria, they were encouraged to be more accurate and/or faster both by written feedback on the screen and verbally by the experimenter. This feedback avoided that participants trading low performance in the processing task for higher performance in the memory task.

##### Processing tasks

*Auditory single-task.* A trial of the auditory single-task (AUD-ST condition) started with a fixation cross-displayed in the centre of the screen for 250 ms. Then randomly either a low- (400 Hz) or high (1000 Hz)-pitched tone was presented for 100 ms via speakers. When the low-pitched tone was presented, participants had to press the “z” key on the keyboard with the left middle finger and when the high-pitched tone was presented, they had to press the “x” key with the left index finger. From its onset, participants had 2000 ms to respond to the stimulus. Participants were instructed to respond as fast and accurately as possible. If participants made a mistake, they received an error message which was displayed for 250 ms (“Error” if an incorrect key was pressed; “Wrong Order” if responses were correct but in the wrong order (only for the dual-task); or “Too Slow” if not responded within 2000 ms). Otherwise a fixation cross was shown for 250 ms. Finally, a fixation cross was shown for a variable duration until the end of the trial (such that each trial in the processing task lasted 2500 ms in total) before moving to the next auditory single-task trial. A processing task consisted of five trials.

*Visual single-task.* A trial in visual single-task (VIS-ST) condition was identical to the auditory single-task except for the following. After the 250 ms fixation cross, instead of a tone, either a blue or yellow circle chosen randomly was presented on the screen for 250 ms. When a blue circle was shown, participants had to press the “left arrow” key on the dedicated arrow-key block between the main keyboard and the keypad/num block with the right index finger and when a yellow circle was presented, the “down arrow” key with the right middle finger.

*Dual-task.* In the dual-task condition (DT), both the auditory and visual stimuli were presented. Three different SOAs were used, 50 ms, 125 ms and 200 ms, which varied randomly within a retention interval. Participants were instructed not to group their responses (Pashler, 1994). After the 250 ms fixation cross, either a high- or low-pitched tone selected randomly was presented and following the variable SOA either a blue or yellow circle was presented. Participants always had to respond first to the auditory and then to the visual stimulus by pressing the same keys as in the respective single-task conditions. The other parameters were the same as described in the single-task conditions. All processing tasks consisted of five trials lasting 2500 ms each.

##### Procedure

The first three participants performed 60 memory trials (10 dual-task memory trials of each of the three memory loads, and 10 single-task (5 auditory and 5 visual) memory trials of each memory load). However, because the experimental run time with this procedure was too long, the remaining 27 participants performed 48 memory trials (8 dual – and 8 single-task memory trials of each memory load). The additional trials of the first three participants are taken into account in the analysis. The order of conditions (memory load and processing task) was individually randomized for each participant. The shortened main experiment lasted about 30 min. Before the main study, participants practiced all tasks for approx. 15 min.

### Results

We assessed the impact of dual-task performance on WM by analysing the recall performance (accuracy) in the memory task. The performance of the single tasks and dual task was measured by response times (RTs) and error rates. In all experiments, we excluded participants if their memory recall for any condition deviated from the respective sample mean data by more than 3 standard deviations. In Experiment 1, no participant was excluded. Eta-squared is used to calculate effect size for paired samples *t* tests according to the following formula:

$$\mathrm{\eta^2}=\frac{{t}^{2}}{({t}^{2}+(N-1)}.$$

#### Recall performance

For the analyses, we calculated the absolute recall performance, which is the proportion of recalled letters in the absolute correct order (Liefooghe et al., 2008). For instance, when presented B, C, D, F and recalling B, C, D, F, the recall score was 4 out of 4 and the recall proportion was 1. However, when B, D, C, F were recalled, the recall score was 2 out of 4 and the recall proportion 0.50, because only the first and last letter matched their serial position in the presentation sequence. This is the standard measure of recall performance for complex WM tasks (Engle, 2002). To test whether dual-task performance impacts WM more than single-task performance (average of VIS-ST and AUD-ST), we compared the recall scores in these two conditions.

**Fig. 4:**
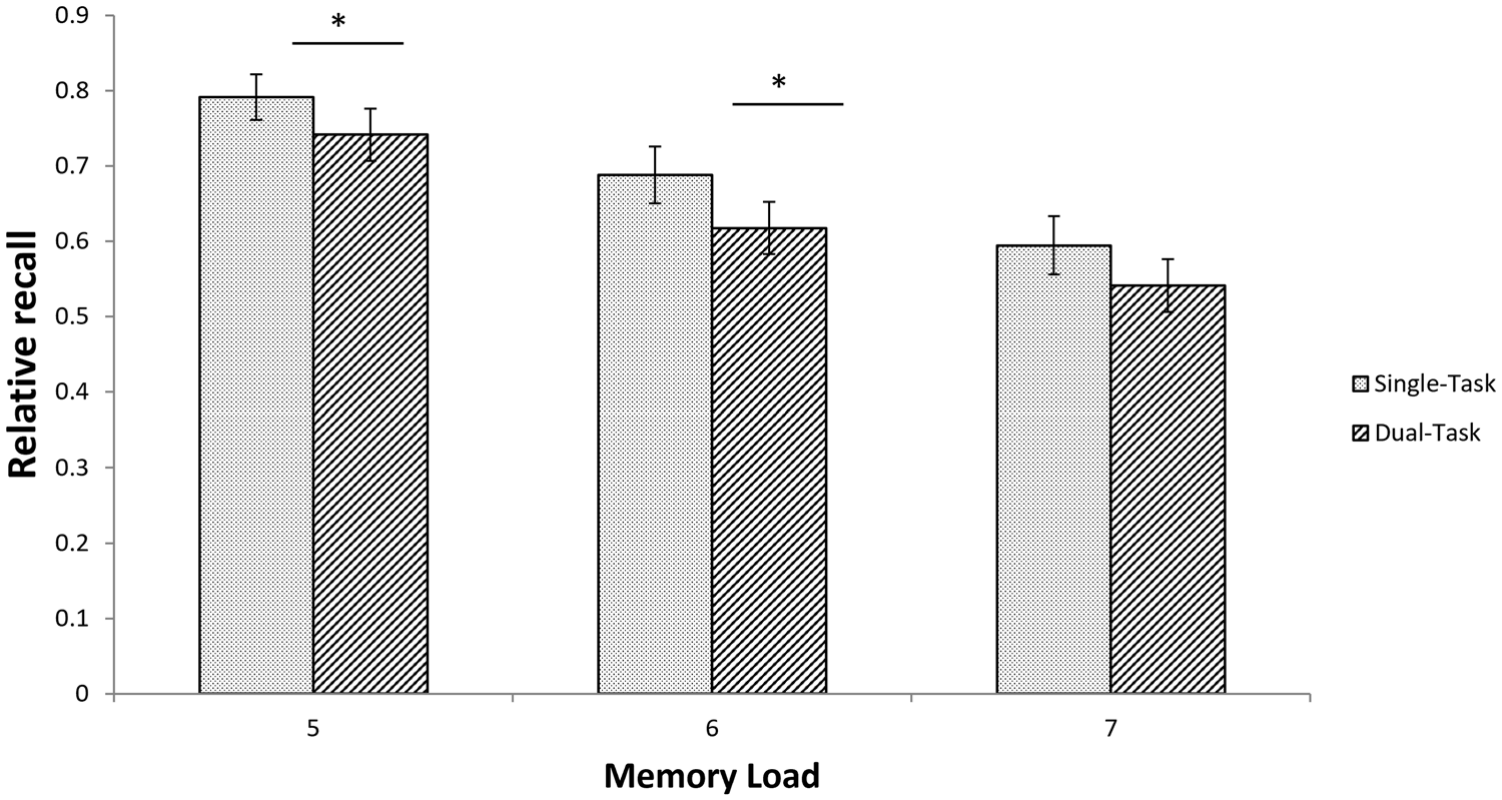
Relative recall performance for single-task and dual-task conditions of Experiment 1 for the three different memory loads. Error bars denote 95% confidence intervals (Loftus & Masson, 1994). Results of paired-sample *t* tests for recall performance differences between single- and dual-task are shown above each pair of bars of memory load (*p < 0.05)

We conducted a 2 × 3 repeated measures ANOVA on the average recall performance with the factors processing task (single-task, dual-task) and memory load (5, 6, 7 letters). In all ANOVA analyses of every experiment, we tested for violations of the sphericity assumption using Mauchly’s test. However, the results of Mauchly’s test are explicitly reported only in case of violations, and in these cases, we report Greenhouse–Geisser corrected statistics. Mauchly’s test indicated that the assumption of sphericity had not been violated for any of the effects. The recall performance was significantly higher in the single-task (0.69 ± 0.17) as compared to the dual-task condition (0.63 ± 0.18) (main effect of processing task: *F*(1, 29) = 10.79, *p* = 0.003, *η*^2^ = 0.27). When analysed separately for each memory load, ST and DT recall significantly differed for load 5 [*t*(1,29) = 2.39, *p* = 0.02, *η*^2^ = 0.16], load 6 [*t*(1,29) = 2.77, *p* < 0.01, *η*^2^ = 0.21], and approached significance for load 7 [*t*(1,29) = 2.03, *p* = 0.051, *η*^2^ = 0.12] (Table 1, Fig. 4), and supports our hypothesis that PRP dual-tasks demand EF of WM. We also analysed the relative recall, i.e. in which all correctly remembered letters are counted irrespective of the order they are presented in and the same pattern of results was observed (i.e., for Experiment 1 higher recall rates if the processing task is a single task as compared to dual task). However, the effects were somewhat smaller, which might be explained by the fact that some participants showed ceiling effects, i.e. 100% correct in single as well as dual tasks. Therefore, we used absolute recall in the rest of the experiments as this a more sensitive measure, at least in our case.

**Table 1:** Relative recall performance of Experiment 1 as a function of processing task and memory load (means ± standard deviation, s.d.)

	Memory load		
Processing task	5	6	7
Single-task	0.79 ± 0.19	0.69 ± 0.18	0.59 ± 0.19
Dual-task	0.74 ± 0.17	0.62 ± 0.20	0.54 ± 0.20

The main effect of memory load was significant [*F*(2, 58) = 44.38, *p* < 0.001, *η*^2^ = 0.61], i.e. recall performance, averaged across processing tasks, was poorer for higher loads. Follow-up paired-sample *t* tests confirmed that all loads differed from each other [all *t*(29) > 3.98, all *p* < 0.001, all *η*^2^ > 0.35]. Processing task and memory load did not interact, *F*(2, 58) = 0.30, *p* = 0.74, *η*^2^ = 0.01.

#### Processing task performance

Performance in the processing tasks was analysed using response times and error rates. In the dual task, participants always had to respond to the auditory stimulus first. This analysis focused on the existence of the PRP effect to show that serial processing occurred, which we proposed requires additional EF for an active scheduling of the tasks. For all dual-task RT analyses, we only used completely error-free trials (no errors in either task 1 or 2).

##### Response times

**Fig. 5:**
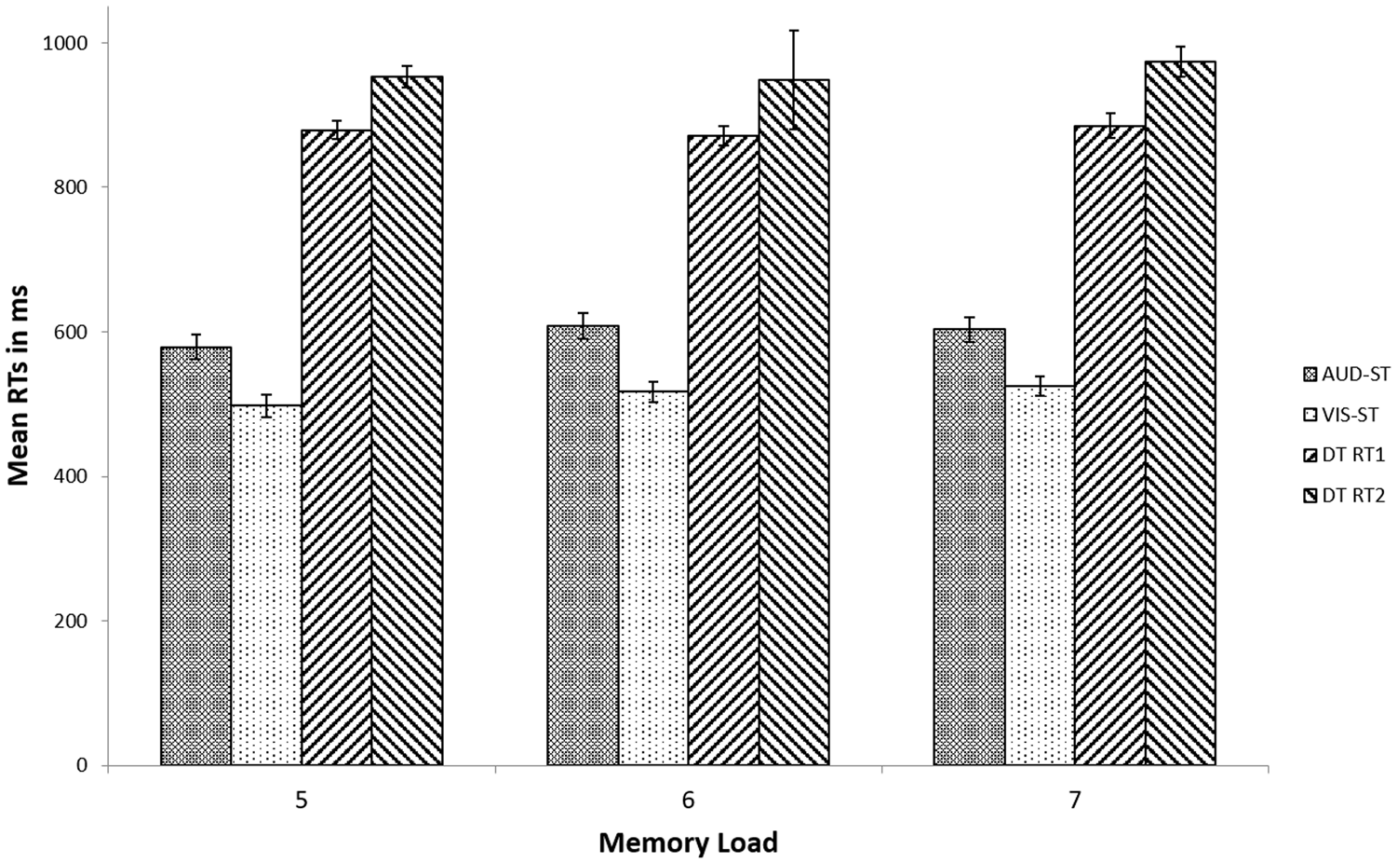
Mean response times for the different processing task conditions per memory load of Experiment 1. In the dual-task, RT1 was always the auditory task, and RT2 the visual task. Error bars denote 95% confidence intervals (Loftus & Masson, 1994)

To provide evidence that a processing bottleneck has been present in the PRP dual-task, we tested for the PRP effect, which is reflected in an increasing RT2 with decreasing SOA while RT1 is rather independent of the SOA. A 2 × 3 repeated measures ANOVA with the factors response (RT1, RT2) and SOA (50, 125 and 200 ms) was conducted to analyse the PRP effect. Analysis revealed a typical PRP effect (Fig. 5). The main effect of response was significant, *F*(1, 29) = 20.89, *p* < 0.001, *η*^2^ = 0.42, showing that on average RT2 is slower than RT1. Also, the main effect of SOA was significant, *F*(2, 58) = 11.26, *p* < 0.001, *η*^2^ = 0.28, showing that on average, response times increased with decreasing SOA. In addition, response and SOA interacted with each other [*F*(2, 58) = 643.75, *p* < 0.001, *η*^2^ = 0.96]. To understand this interaction in more detail, two follow-up one-way repeated measures ANOVAs were conducted for both response times (RT1, RT2) separately. Results showed that the RTs on the second task (RT2) significantly increased with decreasing SOA [*F*(2, 58) = 52.55, *p* < 0.001, *η*^2^ = 0.64], while the RTs of the first task (RT1) remained roughly constant over the range of SOAs [*F*(2, 58) = 2.14, *p* = 0.13, *η*^2^ = 0.07]. Follow-up paired *t* tests showed that RT2 significantly increased with decreasing SOA, from SOA 200 ms to SOA 125 ms [*t*(29) = 6.83, *p* < 0.001, *η*^2^ = 0.62] and from SOA 125 ms to SOA 50 ms [*t*(29) = 4.02, *p* < 0.001, *η*^2^ = 0.36]. Hence, the classic PRP effect was shown for all memory load conditions by prolonged response times to the second stimulus with decreasing SOA, indicating that indeed serial processing occurred in the processing of the PRP dual-task. An overview of the response time per processing task, SOA and memory load is displayed in Table 2 and Fig. 5. Note that the SOA was randomly varied within each retention interval so that it is not possible to analyse the effect of SOA on recall memory performance.

**Table 2:** Response times (ms) and error rates (percentages) of Experiment 1 as a function of processing task and memory load (means ± standard deviation, s.d.)

Task condition	Memory load		
	5	6	7
Dual-task—SOA 50 ms	RT1: 864 ± 203 ms RT2: 1009 ± 218 ms DT1 error: 6.65 ± 7.64% DT2 error: 7.02 ± 8.00%	RT1: 876 ± 170 ms RT2: 1024 ± 195 ms DT1 error: 4.38 ± 5.69% DT2 error: 7.32 ± 9.30%	RT1: 854 ± 171 ms RT2: 1012 ± 181 ms DT1 errror: 5.95 ± 6.15% DT2 error: 7.61 ± 7.38%
Dual-task—SOA 125 ms	RT1: 881 ± 188 ms RT2: 961 ± 212 ms DT1 error: 6.39 ± 5.80% DT2 error: 11.39 ± 8.76%	RT1: 870 ± 203 ms RT2: 952 ± 209 ms DT1 error: 8.26 ± 9.16% DT2 error: 8.17 ± 8.17%	RT1: 908 ± 202 ms RT2: 1003 ± 228 ms DT1 error: 5.76 ± 6.45% DT2 error: 7.39 ± 5.48%
Dual-task—SOA 200 ms	RT1: 885 ± 184 ms RT2: 885 ± 211 ms DT1 error: 9.16 ± 10.74% DT2 error: 9.53 ± 10.28%	RT1: 878 ± 193 ms RT2: 896 ± 210 ms DT1 error: 6.47 ± 8.88% DT2 error: 9.87 ± 9.64%	RT1: 891 ± 198 ms RT2: 902 ± 201 ms DT1 error: 8.56 ± 9.31% DT2 error: 9.05 ± 10.06%
Single-task—AUD	RT1: 579 ± 126 ms Errors: 3.87 ± 4.54%	RT1: 608 ± 138 ms Errors: 3.00 ± 6.16%	RT1: 603 ± 120 ms Errors: 3.63 ± 5.07%
Single-task—VIS	RT1: 498 ± 111 ms Errors: 7.70 ± 6.88%	RT1: 517 ± 101 ms Errors: 7.67 ± 6.98%	RT1: 525 ± 105 ms Errors: 8.93 ± 8.06%

##### Errors

**Fig. 6:**
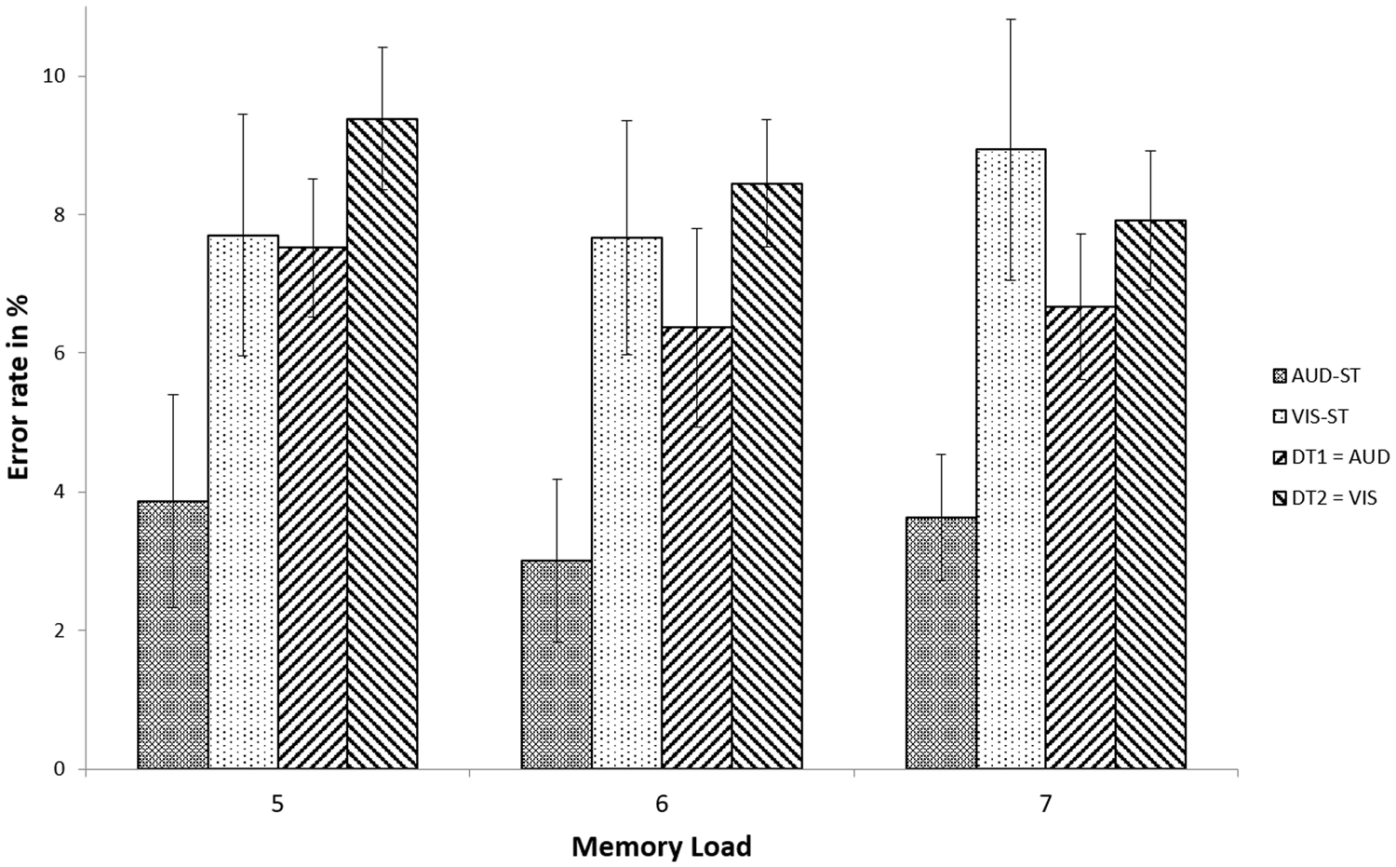
Error rates for the different processing task conditions per memory load of Experiment 1. In the dual-task, RT1 was always the auditory task, and RT2 the visual. Error bars denote 95% confidence intervals (Loftus & Masson, 1994)

We conducted a 2 × 3 repeated measures ANOVA with the factors processing task and memory load to test whether the error rates for the different tasks and loads were different (Table 2, Fig. 6). Specifically, a 2 × 3 repeated measures ANOVA with the factors processing task [single-task auditory, dual-task task 1 (auditory task)] and memory load (5, 6, 7 letters) was conducted to test whether the error rates for the auditory task and loads were different. Results showed that the main effect of memory load was not significant, *F*(2, 58) = 1.00, *p* = 0.37, *η*^2^ = 0.03 indicating that the error rates in the auditory task across loads did not statistically differ from another. The main effect of processing task was significant, *F*(1, 29) = 22.64, *p* < 0.001, *η*^2^ = 0.44. Participants made significantly more errors in the dual-task task 1 (auditory task) (6.85 ± 4.71%) compared to the single-task auditory task (3.50 ± 4.14%). Paired sample *t* tests confirmed this pattern for each memory load [all *t*(29) > 2.64, all *p* < 0.02, all *η*^2^ > 0.19]. This indicates that the dual-task-related RT increases were not due to a speed-accuracy trade-off. Processing task and memory load did not interact, *F*(2, 58) = 0.09, *p* = 0.92, *η*^2^ < 0.001.

Next, the equivalent analysis for the visual task was performed. In more detail, a 2 × 3 repeated measures ANOVA with the factors processing task [single-task visual, dual-task task 2 (visual task)] and memory load (5, 6, 7 letters) was conducted to test whether the error rates for the visual task and loads were different. Results showed that the main effect of memory load was not significant, *F*(2, 58) = 0.17, *p* = 0.85, *η*^2^ = 0.01 indicating that the error rates in the visual task across loads did not statistically differ from another. The main effect of processing task was not significant, *F*(1, 29) = 0.23, *p* = 0.64, *η*^2^ = 0.01. Participants made slightly more errors in the dual-task task 2 (visual task) (8.58 ± 5.21%) compared to the single-task visual task (8.10 ± 5.58%), but this difference was not significant, *t*(29) = 0.48, *p* = 0.64. Processing task and memory load did not interact, *F*(2, 58) = 1.32, *p* = 0.27, *η*^2^ =  < 0.001.

The error rate for the single visual task (8.10 ± 5.58%) was significantly higher than that for the single auditory task (3.50 ± 4.41%), *t*(29) = 4.50, *p* < 0.001. While this may suggest that the visual task might have been more difficult than the auditory task, it is noteworthy that participants responded faster in the visual single-task (513 ± 98 ms) than in the auditory single-task (597 ± 120 ms), *t*(29) = 5.86, *p* < 0.001. Therefore, it cannot be ruled out that the visual and auditory tasks did not differ in their difficulty, but that for some reason, participants adopted different speed-accuracy trade-offs for the two different tasks.

### Discussion

In a complex WM span task in which participants perform a PRP dual-task during the WM retention period, we showed that across all memory loads recall performance was lower after performing a dual-task compared with performing a single task. In addition, we observed a PRP effect, i.e. an SOA-dependent slowing of the second response time, which indicates that the tasks were processed serially for at least one processing stage. The error rate results indicate that participants performed well in the processing tasks. Although there were some significant differences in error rates between single- and dual-task performance, which may indicate that participants somewhat traded off performance in the processing task to increase recall performance, these differences were rather small in terms of effect size.

Our findings show that processing of PRP dual-tasks and WM are related, because dual-task performance affected memory recall more than single-task performance. The TBRS model suggests that this relation is due to the common demands PRP dual-tasks and WM place on the limited resource of controlled attention, i.e. EF (Barrouillet & Camos, 2007). This interpretation is in line with the active scheduling account of bottleneck processing (De Jong, 1995; Luria & Meiran, 2003; Marois & Ivanoff, 2005). Furthermore, our findings suggest that the additional processes involved in coordinating task processing at a serial processing stage, such as inhibition, switching, and monitoring, are closely related or even identical to the EF of WM.

According to the bottleneck model, the first task (in our case always the auditory task) should be unaffected by the presence of the second task. However, the results of Experiment 1 showed that the RTs for the auditory task in the dual-task condition (i.e., DT RT1 in Fig. 5) were longer than the RTs for the auditory task in the single-task condition. The same pattern was observed in the error data of the auditory task. However, as Pashler (1994) has described in detail, it is not uncommon that the first task is also affected during dual-task performance. One potential explanation is so-called response grouping, where participants defer the response to the first task so that they can produce it in a fixed pattern together with the response to the second task.

In this experiment, participants were asked to respond in a certain order to the stimuli, which was constant throughout the block. We believe that this characteristic of the experimental design has no influence on our results and would not affect our interpretation. Even if participants could freely choose in which order to respond, a processing bottleneck and serial processing would still be present. Therefore, EFs would still be demanded to coordinate the serial processing, for example, to inhibit task 2 to avoid interference and to switch the bottleneck between tasks.

## Experiment 2: Order-change manipulation

In Experiment 1, we employed the logic of cognitive subtraction and compared dual- with single-task performance to identify dual-task specific demands on EF. However, in addition to imposing higher demands on EF, dual-task and single-task performance may also differ in other, more global aspects. For example, dual-task performance might impose higher demands on short-term memory when compared with single-task performance. This is because in dual-task blocks participants have to actively maintain two different stimulus–response mappings, or task sets, at the same time (one for each component task) whilst in single-task blocks they only have to maintain one. To circumvent this, Experiment 2 used a parametric manipulation approach in which we compared two dual-task conditions which differed in their demands on controlled attention (cf. Szameitat et al., 2002). In more detail, we manipulated the demands on task-order scheduling by having participants either respond in a constant or in a randomly varying order to the component tasks.

The manipulation is based on findings from De Jong (1995) who showed that the difficulty of a PRP dual-task can be manipulated by varying the order in which the component tasks have to be processed [see also (Luria & Meiran, 2003)]. De Jong (1995) showed that participants automatically prepare to respond in the same order as they did in the previous trial (e.g. AB, AB, AB; A and B denoting the component tasks), and that if the order changes (e.g. AB, BA, AB) response times and error rates are increased.

These behavioural costs in order-change trials arise because participants need to overcome the incorrectly prepared task order. In more detail, in order-change trials, the task which originally was prepared to be processed first needs to be inhibited, and the bottleneck needs to be switched from the expected first task to the actual first task (De Jong, 1995; Luria & Meiran, 2003). Thus, order-change trials impose higher demands on the same EF, i.e. switching and inhibition, which are already involved in the active scheduling of task processing at a bottleneck [cf. (Szameitat et al., 2006)], who observed that order-change and order-repetition trials activated identical brain areas, but to a different strength). Consequently, we predicted that order-change trials impair the concurrent maintenance of items in short-term memory more than same-order trials.

Except for minor changes, Experiment 2 used the same design and stimuli as Experiment 1, i.e. we used a complex WM span task with preload procedure and varied the nature of the processing task which had to be performed during the retention interval. The processing tasks were two dual-task conditions, one with a randomly varying order of the component tasks and one with a fixed task order. We expected recall performance to be lower in the random compared to the fixed processing task.

### Methods

#### Participants

Thirty (28 female) participants (mean age: 19 years, SD = 1.9, range 18–26 years) took part in the study after having given written informed consent. The study was approved by Brunel University’s College of Health and Life Sciences Ethics Committee and participants received course credits for participation.

#### Tasks

The experiment again consisted of a WM span task with preload procedure which consisted of the following phases: cue, memory-encoding phase, retention interval (fixed- or random-order dual-task), recall phase and feedback. In Experiment 2, the two stimuli in the dual-task were always presented simultaneously (with an SOA of 0 ms) and only dual-tasks were used as processing tasks during the retention interval. The phases of Eperiment 2 were the same as Experiment 1 except for the following changes.

##### Block cue

The block cue now instructed the participants about the type of dual-task of the upcoming retention interval processing task and was presented for 4 s. This could be either “Tone -> Colour” or “Colour -> Tone” for the fixed-order conditions or “Random” for the random order condition.

##### Memory-encoding phase

In this experiment, only a memory load of six letters was used.

##### Retention interval

The retention interval lasted 14 s during which participants performed one of the three employed processing tasks, i.e. fixed-order in which participants always had to respond to the auditory (tone) task first (Tone -> Colour), fixed-order in which they always had to respond to the visual (colour) task first (Colour -> Tone), or random order. While only dual-task blocks were used in the main experiment, the single-tasks were used during the practice blocks to familiarize participants with the component tasks.

##### Processing tasks

*Auditory and visual single-task*. The procedure for these tasks was the same as Experiment 1 except that the participant had 2300 ms available to respond instead of 2000 ms.

*Dual-task.* The dual-task conditions were the same as in Experiment 1, except for the following changes. First, we added the random order dual-task condition in which participants had to respond to the tasks in a randomly changing order, as instructed by a cue in each trial. Second, because this condition was more difficult, participants now had 2300 ms to respond to the stimuli. Third, we used a single SOA of 0 ms. Finally, to instruct participants in which order they had to respond to the stimuli, in the random-order condition, a cue was presented in each trial with the visual stimulus.

This cue was either a square or a diamond placed around the circle. In case of a square, participants had to respond first to the tone and then to the colour of the circle, whereas in case of a diamond, participants first had to respond to the colour of the circle and then to the tone. There were 3 different order conditions in the dual task, and participants were always presented five trials in each retention interval (lasting 14 s). The first condition (“Tone -> Colour”) consisted of a constant auditory–visual order in which participants first had to respond to the tone and then to the colour of the circle. The second condition (“Colour -> Tone”) was a constant visual–auditory block in which participants always had to respond first to the colour of the circle followed by responding to the tone. The third condition (“Random”) consisted of a random task block in which the occurrence of the two different orders was randomized with the restriction that at least two order-changes had to be present in each processing task consisting of five trials. Note that all conditions were identical, except for the instructions (block cue) and the task cue. All processing tasks consisted of five trials lasting 2800 ms each.

##### Procedure

There was one memory load (six letters) and three different types of dual task in the retention interval. Pilot studies (not reported) using the same methodology had shown that participants find it difficult to adjust to the different dual-task conditions. This became clear by a post-study questionnaire that the participants filled in and it was also mentioned by each participant to the researcher verbally. Therefore, all memory trials were presented in pairs, e.g. “Random”, “Random”, “Tone -> Colour”, “Tone -> Colour”, etc. so that participants had one memory trial to adjust to the respective condition. The order of conditions was pseudo-randomized, with the just mentioned restriction that conditions were always presented in pairs. In total, each participant performed 36 memory trials (12 dual-task memory trials of each of the three dual-task conditions). The main experiment lasted about 25 min. Before this, participants practiced all tasks (including the single tasks) for approx. 25 min.

### Results

Unlike in the pilot studies, participants had no difficulties adjusting to the different tasks, so all blocks were included in the analysis (except for the first 4 participants, for whom we obtained data only for the second block of each pair due to a programming error). Again, the impact of dual-task performance on WM was analysed by the recall performance in the memory task. The performance of the dual tasks was again measured by response times and error rates, and only completely error-free trials were used for the response time analyses. No participants were excluded. In these analyses, the fixed condition is the average of the “Tone -> Colour” and “Colour -> Tone” conditions.

#### Recall performance

To test whether the order manipulation had an effect on memory recall, we compared the recall performance between the fixed and random processing task conditions using paired-sample *t* tests. The recall performance was significantly higher in the fixed (0.58 ± 0.15) as compared to the random condition (0.51 ± 0.21), *t*(29) = 3.00, *p* < 0.01, *η*^2^ = 0.24 supporting the hypothesis that higher demands on EF in a dual task adversely affects memory performance. Recall performance of the two fixed conditions, “Tone -> Colour” (0.58 ± 0.16) and “Colour -> Tone” (0.58 ± 0.16), did not significantly differ from each other, *t*(29) = 0.12, *p* = 0.91, *η*^2^ = 0.00.

#### Processing task performance

##### Response times

We conducted a 2 × 2 repeated measures ANOVA with the factors response (RT1, RT2) and processing task (fixed, random) to analyse the response times for both task types; Table 3. Response times to the second task were significant slower than those of the first task [main effect of response *F*(1, 29) = 306.98, *p* < 0.001, *η*^2^ = 0.91]. This effect was present in the fixed and the random order task conditions, all *t*(29) > 16.48, all *p* < 0.001, all *η*^2^ > 0.90. The main effect of processing task was significant, *F*(1, 29) = 221.19, *p* < 0.001, *η*^2^ = 0.88. Follow-up *t* tests showed that both the RT1s (1286 ± 154 ms) and RT2s (1564 ± 157 ms) of the random condition were significantly higher than the RT1s (1027 ± 135 ms) and RT2s (1297 ± 147 ms) of the fixed condition, respectively [all *t*(29) > 13.90, all *p* < 0.001, all *η*^2^ > 0.86]. Response and processing task did not interact, *F*(1, 29) = 2.33, *p* = 0.14, *η*^2^ = 0.07.

**Table 3:** Response times (ms) and error rates (percentages) of each dual-task condition of Experiment 2

Processing task	RT in ms		Error rate in %	
	RT1	RT2	Task1	Task2
Fixed	1027 ± 135	1297 ± 147	6.86 ± 7.83	9.61 ± 8.46
Random	1286 ± 154	1564 ± 157	13.22 ± 11.79	15.39 ± 12.80

##### Error rates

We conducted a 2 × 2 repeated measures ANOVA with the factors task error (task1, task2) and processing task (fixed, random) to analyse the error rates for both task types; Table 3. These task errors in which multiple errors can occur in principle, were considered either as correct or erroneous, irrespective of the number of errors made (i.e. a wrong response or responded too late). The main effect of processing task was significant, *F*(1, 29) = 44.23, *p* < 0.001, *η*^2^ = 0.60. Follow-up paired-samples *t* tests showed that both the errors in task 1 (13.22 ± 11.79%) and in task 2 (15.39 ± 12.80%) of the random condition were significantly higher than the errors in task 1 (6.86 ± 7.83%) and in task 2 (9.61 ± 8.46%) of the fixed condition, respectively [all *t(*29) > 5.59, all *p* < 0.001, all *η*^2^ > 0.51]. The main effect of task error was also significant, *F*(1, 29) = 11.34, *p* = 0.002, *η*^2^ = 0.28. Follow-up paired samples *t* tests showed that the error in task 1 was significantly lower than the errors in task 2 for both the fixed and random conditions [all *t*(29) > 2.07, all *p* < 0.05, all *η*^2^ > 0.13]. Task error and processing task did not interact, *F*(1, 29) = 0.39, *p* = 0.54, *η*^2^ = 0.01. Participants could also respond in the wrong order and this type of error was significantly larger in the random condition (15.44 ± 13.17%) compared to the fixed condition (5.14 ± 5.18%), *t*(29) = 7.00, *p* < 0.001, *η*^2^ = 0.63.

### Discussion

Experiment 2 showed that recall performance was lower after performing a difficult dual task (random-order condition) during the retention interval when compared with an easier dual task (fixed-order condition). In the random order condition, the error rate was higher [similar error rates were reported in Szameitat et al., (2002)] and the RT2 was longer compared to the fixed condition and so the random condition can be considered to be a highly difficult task.

The current findings support and extend the conclusions from Experiment 1 that the processing of PRP dual-tasks is related to WM. Experiment 2 confirmed that this relationship is not simply due to higher memory demands in the dual-task as compared with the single-tasks, or other potentially confounding differences between single- and dual-task performance. Instead, the relationship seems to arise from the demands the tasks place on common processes, i.e. controlled attention (Barrouillet & Camos, 2007). Specifically, scheduling the order in which the tasks are processed at the stage of a bottleneck, which involves switching and inhibition, places demands on the same mental resources as short-term memory maintenance. Thus, the current study provided further support for the hypothesis that the processes which actively schedule tasks at a bottleneck are related to or are even identical to the EF of WM.

In this study, both component tasks were always presented at the same time, i.e. SOA of 0 ms, so that we could not assess a PRP effect as evidence for the occurrence of serial processing. However, only minor changes were made to Experiment 1, in which we did observe the PRP effect, so that we believe that a bottleneck and serial processing was present in this experiment as well. In line with this, the response times of the second task in this experiment were comparable or even longer than in Experiment 1 and also considerably longer than those of the single tasks in experiment 1, which points to the typical deferment of the second task. However, future studies could manipulate the SOA and the response order independently.

## Experiment 3: Order-change frequency

When scrutinizing the two dual-task conditions in Experiment 2 in more detail, it appears that they might not only differ in their demands on task-order coordination, but additionally in their demands on cue processing. The cue, which informed participants about the order in which they had to respond to the component tasks (a square or diamond around the circle), was presented only in the random-order condition, but not in the fixed-order condition. Participants likely kept the cue meanings in their short-term memory so that the random-order condition may have imposed higher memory demands, which presents an alternative explanation for the findings of Experiment 2. To rule out this alternative explanation, we conducted a further experiment in which we presented two random-order conditions, one with a low and one with a high number of order-change trials. In both cases, participants needed to maintain cue meaning and process the cue. If PRP dual-tasks are related to the EF of WM, a condition with a high number of order-changes should result in poorer memory recall as compared to a condition with a low number of order-changes.

### Methods

#### Participants

Thirty-four (29 female) participants (mean age: 20 years, SD = 5.5, range 18–46 years) took part in the study after having given written informed consent. The study was approved by Brunel University’s College of Health and Life Sciences Ethics Committee and participants received course credits for participation.

#### Tasks

The experiment again consisted of a complex WM span task with preload procedure which consisted of the following phases: cue, memory-encoding phase, retention interval (low or high switches dual-task), recall phase and feedback. The phases of Experiment 3 were the same as Experiment 2 except for the following changes.

##### Block Cue

The block cue now instructed the participants about the type of upcoming dual task, which could be either “Low Switches” or “High Switches”.

##### Retention interval

The retention interval lasted longer, 35.2 s, during which participants performed one of the two employed processing tasks, i.e. random low order-changes or random high order-changes. The retention interval needed to be longer to present more dual-task trials to implement two conditions with high and low frequencies of order-change trials. Pilot testing confirmed that participants are able to perform the task, in particular the memory retention, with the extended retention interval.

##### Processing tasks.

*Auditory and visual single-task.* The procedure for these tasks, which were used only during practice, was the same as Experiment 2 except that the participant had 2700 ms to respond instead of 2300 ms.

*Dual-task.* The dual-task conditions were the same as in Experiment 2, except for the following changes. Again, the cue indicated the order in which the participant needed to respond to the stimuli, a diamond for colour first and a square for tone first. Participants had 2700 ms to respond to both stimuli. There were 2 different order conditions (low and high number of order-changes) in the dual task. Each retention interval consisted of 11 trials, each lasting 3200 ms. In the low order-changes condition, there were always 2 order-change trials, i.e. in two out of the eleven trials, participants had to change the order in which they had to respond to the two tasks, as instructed by the cue. In the high order-change condition, there were always 9 order-changes in any retention interval, i.e. in nine out of the eleven trials, participants had to change the order in which they had to respond to the tasks. The order-change trials occurred randomly in each block.

##### Procedure

There was only one memory load (6 letters) and 2 types of dual-task. In total, each participant performed 20 memory trials (10 per dual-task condition). The main experiment lasted about 20 min. Before this, participants practiced all tasks (including the single-tasks) for approx. 30 min, which included a thorough practice of the quite challenging random dual-tasks.

### Results

Again, the impact of dual-task performance on WM was analysed by the recall performance in the memory task. The performance of the dual-tasks was again measured by response times and error rates. One participant was classified as an outlier (mean difference in recall between the low and high order-changes was more than 3 standard deviations from the group mean difference) and hence excluded from the analysis.

#### Recall performance

To test whether the order-change manipulation had an effect on memory recall, we compared the recall performance between the low and high order-change processing task conditions using a paired-sample *t* test. The recall performance was significantly higher in the low order-change (0.57 ± 0.21) as compared to the high order-change condition (0.54 ± 0.19), *t*(32) = 2.11, *p* = 0.04, *η*^2^ = 0.12. This supports the hypothesis that the difficulty of a dual-task adversely affects memory performance.

#### Processing task performance

##### Response times

We conducted a 2 × 2 repeated-measures ANOVA with factors response (RT1, RT2) and processing task (low and high order-changes) to analyse the response time for both order-change task conditions; Table 4. The main effect of processing task was significant [*F*(1, 32) = 60.57 *p* < 0.001, *η*^2^ = 0.65]. Follow-up *t* tests showed that both the RT1s (1452 ± 171 ms) and RT2s (1736 ± 217 ms) of the high order-change condition were significantly larger than the RT1s (1320 ± 138 ms) and the RT2s (1610 ± 176 ms) of the low order-change condition, respectively [all *t*(32) > 7.45, all *p* < 0.001, all *η*^2^ > 0.63]. The main effect of response was significant [*F*(1, 32) = 214.44, *p* < 0.001, *η*^2^ = 0.88]. Follow-up *t* tests showed that for both order-change task conditions, the RT2s were significantly longer than their respective RT1s [all *t*(32) > 15.31, all *p* < 0.001, all *η*^2^ > 0.87]. There was no interaction between response and processing task *F*(1, 32) = 0.93, *p* = 0.34, *η*^2^ = 0.03.

**Table 4:** Response times (ms) and error rates (percentages) of each dual-task condition of Experiment 3

Processing task	RT in ms		Error rate in %	
	RT1	RT2	Task1	Task2
Low order-change	1320 ± 138	1610 ± 176	9.92 ± 8.56	13.61 ± 9.55
High order-change	1452 ± 171	1736 ± 217	14.30 ± 11.00	18.48 ± 12.49

##### Error rates

We conducted a 2 × 2 repeated measures ANOVA with the factors task error (task1, task2) and processing task (low and high order-changes) to analyse the error rates for both task types; Table 4. The main effect of processing task was significant, *F*(1, 32) = 20.09, *p* < 0.001, *η*^2^ = 0.39. Follow-up paired-samples *t* tests showed that both the errors in task 1 (14.30 ± 11.00) and in task 2 (18.48 ± 12.49) of the high order-change condition were significantly higher than the errors in task 1 (9.92 ± 8.56) and in task 2 (13.61 ± 9.55) of the low order-change condition, respectively (all *t(*29) > 4.09, all *p* < 0.001, all *η*^2^ > 0.34). The main effect of task error was also significant, *F*(1, 29) = 22.69, *p* < 0.001, *η*^2^ = 0.42. Follow-up paired samples *t* tests showed that the error in task 1 was significantly lower than the errors in task 2 for both the fixed and random conditions [all *t*(29) > 4.04, all *p* < 0.001, all *η*^2^ > 0.33]. Task error and processing task did not interact, *F*(1, 29) = 0.33, *p* = 0.57, *η*^2^ = 0.01. Participants could also respond in the wrong order and this type of error was significantly larger in the high order-change condition (8.18 ± 8.19) compared to the low order-change condition (4.79 ± 5.66), *t*(29) = 3.53, *p* < 0.002, *η*^2^ = 0.28.

### Discussion

Experiment 3 supported and refined the findings of Experiment 2 by showing that memory performance is lower when participants have to perform a high number of order-changes during the retention interval as compared to a low number of order-changes. Experiment 3 ruled out the alternative explanation that a difference in memory load, caused by the task cue, might account for the observed differences between fixed and random order conditions in Experiment 2. Such differences were absent in the current experiment, because both conditions required the processing of the cue. Based on the TBRS model, we suggest that instead both tasks, i.e. (a) PRP dual-tasks with active scheduling of serial processing and (b) memory maintenance, demand controlled attention, i.e. EF. Consequently, we conclude that PRP dual-tasks do demand the EF of WM beyond the demands imposed by the sole performance of the single-tasks. We would like to note that this experiment was replicated in the context of a student project with a new sample consisting of 46 (39 female) participants (mean age: 20 years, SD = 1.3, range 18–23 years) and similar results were found: The recall performance was significantly higher in the low order-change condition (0.55 ± 0.26) as compared to the high order-change condition (0.50 ± 0.26), *t*(45) = 2.37, *p* = 0.02, *η*^2^ = 0.16.

## General discussion

In the present study, we investigated the nature of the mental processes in dual-task processing involved in coordinating serial task processing. More specifically, we aimed to clarify whether these processes are related to the EF of WM. This was investigated using a complex WM span task that combined the PRP paradigm with a short-term memory task. In more detail, participants had to memorize letters (memory task) and perform various processing tasks (single-tasks and dual-tasks of varying difficulty) during the retention interval before recalling the initially memorized letters in their order of presentation. In this complex WM span task participants have to manage concurrent demands on short-term memory maintenance and processing of information. These two demands, maintenance and processing, are at the heart of many influential WM models (Baddeley & Hitch, 1974; Barrouillet & Camos, 2007; Engle, 2002; Oberauer & Lewandowsky, 2013). Consequently, if the presence of serial processing in dual-tasks affects short-term memory maintenance, this is strong evidence that both rely at least in part on the same mental mechanisms, i.e. the EF of WM.

Experiment 1 showed that participants have poorer recall performance after performing a dual-task compared with a single task during the WM retention interval, and this effect was present in all three different memory loads. Experiments 2 and 3 were performed to show the effect of Experiment 1 whilst using a parametric modulation of dual-task demands and, therefore, to explore the WM demands of multitasking further. In more detail, Experiment 2 investigated the role of task-order coordination, which has been shown to be one fundamental aspect of task demands in PRP dual-tasks (De Jong, 1995; Kübler et al., 2018; Luria & Meiran, 2003; Szameitat et al., 2006). For this, participants either had to respond in the same order to the two tasks in subsequent trials (fixed condition), or in a randomly changing order (random condition). Results showed poorer recall performance in the random as compared to the fixed condition, indicating that increased demands on task-order scheduling in PRP dual-tasks result in increased demands on the EF of WM. Experiment 3 investigated this finding in even more detail, by comparing blocks with a high number of task-order changes to blocks with a low number of task-order switches. Results showed reduced recall performance with high numbers of task-order switches and, therefore, that EF of WM are subject to increased demand. All these experiments show that the performance of a PRP dual-task places increased demands on the EF of WM, most likely because of the presence of a central attentional bottleneck.

The above findings have implications for theoretical models of dual-task performance. Two competing theories have been proposed, the active scheduling account which postulates that additional control processes are required to coordinate serial task processing at the bottleneck (De Jong, 1995), and the passive queuing account, which postulates that the tasks are processed by the bottleneck passively on a first-come first-served basis so that no additional processes are demanded (Jiang et al., 2004). Our findings are in clear support of the active scheduling account. In particular, Experiment 3 showed that increasing the task-order demands of a dual-task leads to increased impairments in memory recall. The passive queuing account does not propose task-order coordination mechanisms and, therefore, cannot explain the current findings. Of course, the mere fact that participants can adjust the processing order of the tasks based on an explicit task-cue with a constant SOA of 0 ms already shows that some form of control mechanisms must be at work. This is in line with previous research which also supported the active scheduling account. For instance, De Jong (1995) and Luria and Meiran (2003) showed that participants have explicit control over the processing order of the tasks and can utilize advance cue information to prepare processing order. In addition, evidence from functional neuroimaging has shown that bottleneck processing results in additional brain activation which goes beyond a mere summation of the brain activity elicited by both single tasks (Dux et al., 2006; Marois & Ivanoff, 2005; Szameitat et al., 2002). This additional brain activation is an indicator of additional mental processing. We interpret this additional mental processing as the processing linked to the active scheduling demands. Thus, although some research questions the active scheduling account (Jiang et al., 2004), it seems that the majority of research, including the current study, is in support of it.

The major contribution of the current study, however, lies in the characterization of the additional processes required to coordinate serial processing in PRP dual-tasks. Previous empirical and theoretical work suggested that serial processing demands processes, such as inhibition (of task 2 to avoid interference between the tasks) and switching (of the serial processing stage from task 1 to task 2). Although the terms inhibition and switching are also prototypical functions of EF (Miyake et al., 2000), it was unclear whether the task switching and working memory research communities just used the same terminology, or whether they indeed referred to the same mental mechanisms. In other words, does serial processing demand the EF of WM, or does it demand some other mental mechanisms which may have similar functionality but are distinct from the EF of WM? Our findings resolved this question and show that PRP dual-tasks demand the EF of WM. This finding opens up new perspectives on the active scheduling account and may inspire more research on the links between processes controlling human action and processes controlling human WM.

Our conclusion that serial processing demands the EF of WM is in line with previous evidence derived from functional neuroimaging. As mentioned above, performing a PRP dual-task results in additional brain activations which cannot be explained by the summed activations of the single-tasks. Noteworthy, this additional brain activation is localized in a fronto-parietal network of brain areas well known to subserve EF (Szameitat et al., 2002). Furthermore, research has shown that participants with impaired EF (highly neurotics) show correlating changes in dual-task-related brain activity (lateral, and medial prefrontal cortices) and dual-task costs (Szameitat et al., 2016). Thus, data from functional neuroimaging and behavioural experiments converge on the conclusion that serial processing in PRP-dual-tasks demands the EF of WM.

The current findings have implications for the interpretation of response time effects in the PRP paradigm. Usually, the deferment of the second task due to the serial processing stage being blocked by the first task, i.e. the PRP effect, has been attributed to be a mere waiting time (or slack). While rarely spelled out explicitly, this implicitly assumes a passive queuing account. However, it seems implausible to assume that the EF coordinating the serial processing would not affect the response times, and indeed De Jong (1995) provided some indications that control processes contribute to the PRP effect. Consequently, the PRP effect is likely to be a mixture of a purely passive waiting time (slack) and a deferment of task processes due to EF actively coordinating the processing, such as switching the serial processing stage to the second task after it has finished processing the first task. The EF may also account for the not uncommon finding in the PRP task that the response times of the first task are also prolonged, which is not directly predicted by a pure PRP model (De Jong, 1995; but see Pashler, 1994 for alternative explanations).

As explained above, several executive functions have been postulated to be potentially involved in the coordination of the two tasks in PRP dual-tasks, such as inhibition of task 2 to avoid interference, switching of the bottleneck between tasks, or task-order scheduling. The current findings do not allow to identify the exact function which may interact with the working memory span task, it could be just one or any combination of them.

## Conclusion

To conclude, the presence of serial processing in multitasking imposes additional processing demands beyond the mere sum of those imposed by the single-task performance. Furthermore, we showed that these additional mental processes are tightly linked, if not identical, to the executive functions of working memory.

## Data Availability

Data can be downloaded from Brunel figshare repository. The following link is a private link that can be used for the reviewers/editors if they wish to view the data. This link does not contain my name or institute and is suitable for (double blind) peer review: https://www.figshare.com/s/e20831dfabf7236e3620. This link is suitable for 2 years, but will not be included in the final data availability statement. This link will be updated with the DOI for anyone to see once the article is published.

## Code availability

Not applicable. The experiment was programmed in E-Prime 2, the code can be made available upon request.

## References

[CR1] Baddeley AD (1996). Exploring the central executive. The Quarterly Journal of Experimental Psychology Section A.

[CR2] Baddeley AD, Hitch G (1974). Working memory. The psychology of learning and motivation: Advances in research and theory.

[CR3] Barrouillet, P., & Camos, V. (2007). The time-based resource-sharing model of working memory. In N. Osaka, R.H. Logie, M. D'Esposito (Eds.), *The cognitive neuroscience of working memory* (pp. 59–80). Oxford University Press.

[CR4] Barrouillet P, Camos V (2010). Working memory and executive control: A time-based resource-sharing account (1). Psychologica Belgica.

[CR301] Bavelier, D., Newport, E. L., Hall, M. L., Supalla, T., & Boutla, M. (2006). Persistent difference in short-term memory span between sign and speech: Implications for cross-linguistic comparisons. *Psychological Science,**17*(12), 1090–1092. 10.1111/j.1467-9280.2006.01831.x 17201792

[CR300] Crannell, C. W., & Parrish, J. M. (1957). A comparison of immediate memory span for digits, letters, and words. *The Journal of Psychology,**44*(2), 319–327.

[CR5] De Jong R (1995). The role of preparation in overlapping-task performance. The Quarterly Journal of Experimental Psychology Section A.

[CR6] Dux PE, Ivanoff J, Asplund CL, Marois R (2006). Isolation of a central bottleneck of information processing with time-resolved fMRI. Neuron.

[CR7] Engle RW (2002). Working memory capacity as executive attention. Psychological Science.

[CR8] Engle, R. W., Kane, M. J., & Tuholski, S. W. (1999). Individual differences in working memory capacity and what they tell us about controlled attention, general fluid intelligence, and functions of the prefrontal cortex. In A. Miyake & P. Shah (Eds.), *Models of working memory* (pp. 102–134). Cambridge University Press. 10.1017/CBO9781139174909.007

[CR9] Fischer R, Plessow F (2015). Efficient multitasking: Parallel versus serial processing of multiple tasks. Frontiers in Psychology.

[CR10] Jiang Y, Saxe R, Kanwisher N (2004). Functional magnetic resonance imaging provides new constraints on theories of the psychological refractory period. Psychological Science.

[CR11] Koch I, Poljac E, Müller H, Kiesel A (2018). Cognitive structure, flexibility, and plasticity in human multitasking-an integrative review of dual-task and task-switching research. Psychological Bulletin.

[CR12] Kübler S, Reimer CB, Strobach T, Schubert T (2018). The impact of free-order and sequential-order instructions on task-order regulation in dual tasks. Psychological Research Psychologische Forschung.

[CR13] Kübler, S., Strobach, T., & Schubert, T. (2021). The role of working memory for task-order coordination in dual-task situations. *Psychological Research*, 1–22. Published online 10.1007/s00426-021-01517-2 PMC8885531 33884485

[CR14] Lee JJ, Chabris CF (2013). General cognitive ability and the psychological refractory period: Individual differences in the mind’s bottleneck. Psychological Science.

[CR15] Lehle C, Hübner R (2009). Strategic capacity sharing between two tasks: Evidence from tasks with the same and with different task sets. Psychological Research Psychologische Forschung.

[CR16] Liefooghe B, Barrouillet P, Vandierendonck A, Camos V (2008). Working memory costs of task switching. Journal of Experimental Psychology: Learning, Memory, and Cognition.

[CR17] Logan GD, Gordon RD (2001). Executive control of visual attention in dual-task situations. Psychological Review.

[CR18] Luria R, Meiran N (2003). Online order control in the psychological refractory period paradigm. Journal of Experimental Psychology: Human Perception and Performance.

[CR19] Marois R, Ivanoff J (2005). Capacity limits of information processing in the brain. Trends in Cognitive Sciences.

[CR20] Marois R, Larson JM, Chun MM, Shima D (2006). Response-specific sources of dual-task interference in human pre-motor cortex. Psychological Research Psychologische Forschung.

[CR21] Meyer DE, Kieras DE (1997). A computational theory of executive cognitive processes and multiple-task performance: Part I. Basic Mechanisms. Psychological Review.

[CR22] Miyake A, Friedman NP, Emerson MJ, Witzki AH, Howerter A, Wager TD (2000). The unity and diversity of executive functions and their contributions to complex “frontal lobe” tasks: a latent variable analysis. Cognitive Psychology.

[CR23] Oberauer K, Lewandowsky S (2013). Evidence against decay in verbal working memory. Journal of Experimental Psychology: General.

[CR24] Pashler H (1990). Do response modality effects support multiprocessor models of divided attention?. Journal of Experimental Psychology: Human Perception and Performance.

[CR25] Pashler H (1993). Doing two things at the same time. American Scientist.

[CR26] Pashler H (1994). Dual-task interference in simple tasks: Data and theory. Psychological Bulletin.

[CR27] Schubert T (2008). The central attentional limitation and executive control. Frontiers in Bioscience: A Journal and Virtual Library.

[CR29] Sigman M, Dehaene S (2005). Parsing a cognitive task: A characterization of the mind’s bottleneck. PLoS Biology.

[CR30] Smith MC (1967). Theories of the psychological refractory period. Psychological Bulletin.

[CR31] Souza AS, Oberauer K (2015). Time-based forgetting in visual working memory reflects temporal distinctiveness, not decay. Psychonomic Bulletin & Review.

[CR32] Spence C (2008). Cognitive neuroscience: Searching for the bottleneck in the brain. Current Biology.

[CR33] Szameitat AJ, Lepsien J, Von Cramon DY, Sterr A, Schubert T (2006). Task-order coordination in dual-task performance and the lateral prefrontal cortex: An event-related fMRI study. Psychological Research Psychologische Forschung.

[CR34] Szameitat AJ, Saylik R, Parton A (2016). Neuroticism related differences in the functional neuroanatomical correlates of multitasking. An fMRI Study. Neuroscience Letters.

[CR35] Szameitat AJ, Schubert T, Müller K, von Cramon DY (2002). Localization of executive functions in dual-task performance with fMRI. Journal of Cognitive Neuroscience.

[CR36] Tombu M, Jolicœur P (2004). Virtually no evidence for virtually perfect time-sharing. Journal of Experimental Psychology: Human Perception and Performance.

[CR37] Tombu MN, Asplund CL, Dux PE, Godwin D, Martin JW, Marois R (2011). A Unified attentional bottleneck in the human brain. Proceedings of the National Academy of Sciences.

[CR38] Unsworth N, Heitz RP, Schrock JC, Engle RW (2005). An automated version of the operation span task. Behavior Research Methods.

